# Associations between body mass index and foot joint pain in middle-aged and older women: a retrospective analysis of a longitudinal cohort

**DOI:** 10.1186/1757-1146-7-S2-A4

**Published:** 2014-11-14

**Authors:** Anita Gay, Catherine Bowen, David Culliford, Nigel Arden

**Affiliations:** 1 Faculty of Health Sciences, University of Southampton, Southampton, UK; 2 Nuffield Department of Orthopaedics, Rheumatology and Musculoskeletal Sciences, University of Oxford, Oxford, UK; 3 Faculty of Medicine, University of Southampton, Southampton, UK

## Objectives

The objective of this study was to determine if a high body mass index (BMI) predicts foot joint pain (FJP) in middle-aged and older women over a 5-year period.

## Methods

A retrospective, longitudinal, cohort study design was used to investigate the relationship between patient reported foot joint pain (FJP), body mass index (BMI) and age over time. Data has been prospectively collated (20 years) for women from the general population, the ‘1000 Women Study’. From a baseline of 1003 female participants, data from 639 women (64%) were reviewed at years (Y) 10 and 15.

## Results

For year 10 and 15 (respectively) the median age was 61 years (57-67), 66 years (62-72); mean BMI 26.7 kg/m2 (± 4.6), 27.2 kg/m2 (4.8). BMI increased significantly from Y10 to Y15 (p < 0.001). The FJP prevalence was 21.6% at year10 and 26.6% year15. The odds of having FJP after a 5-year period increased by 4.9% for each BMI unit increase 5 years earlier (OR 1.049, 95% CI 1.011-1.089; p = 0.012). This remained significant when adjusted for age (OR 1.049, 95% CI 1.011-1.089; p = 0.012). A previous episode of FJP was a stronger predictor of having FJP 5 years later (OR 3.678, 95% CI 2.465-5.489; p < 0.001).

## Conclusion

This study confirms that a high BMI is likely to predict FJP in middle-aged women. These findings provide additional evidence to identify patients at risk of developing FJP, as well as evidence that foot health clinicians have a key role in public health interventions related to obesity.

